# Prediction of Prognosis and Recurrence of Bladder Cancer by ECM-Related Genes

**DOI:** 10.1155/2022/1793005

**Published:** 2022-04-12

**Authors:** Hongfan Zhao, Zihao Chen, Yunze Fang, Mingqiang Su, Yipeng Xu, Zhifeng Wang, Michael Adu Gyamfi, Junfeng Zhao

**Affiliations:** ^1^Department of Urology, Southern Medical University, Guangzhou, China; ^2^Department of Urology, The Cancer Hospital of the University of Chinese Academy of Sciences (Zhejiang Cancer Hospital), Hangzhou, China; ^3^Department of Urology, Henan Provincial People's Hospital, Zhengzhou University People's Hospital, Zhengzhou, China; ^4^Department of Biomedical Sciences, University of Health and Allied Sciences, Ho, Ghana; ^5^Department of Urology, The Second Affiliated Hospital of Henan University of Traditional Chinese Medicine, Zhengzhou, China

## Abstract

**Background:**

Bladder cancer (BLCA) is one of the most common cancers and ranks ninth among all cancers. Extracellular matrix (ECM) genes activate a number of pathways that facilitate tumor development. This study is aimed at providing models to predict BLCA survival and recurrence by ECM genes.

**Methods:**

Expression data from BLCA samples in GSE32894, GSE13507, GSE31684, GSE32548, and TCGA-BLCA cohorts were downloaded and analyzed. The ECM-related genes were obtained by differentially expressed gene analysis, stage-associated gene analysis, and random forest variable selection. The ECM was constructed in GSE32894 by the hub ECM-related genes and validated in GSE13507, GSE31684, GSE32548, and TCGA-BLCA cohorts. The correlations of the ECM score with cells (T cells, fibroblasts, etc.) and the response to immunotherapeutic drugs were investigated. Four machine learning models were selected and used to construct models to predict the recurrence of BLCA. A total of 15 paired BLCA and normal tissue specimens, human immortalized uroepithelial cell lines, and bladder cancer cell lines were selected for the validation of the difference in expression of FSTL1 between normal tissues and BLCA.

**Results:**

Six ECM genes (CTHRC1, MMP11, COL10A1, FSTL1, SULF1, and COL5A3) were recognized to be the hub ECM-related genes. The ECM score of each BLCA patient was calculated using these six selected ECM-related genes. BLCA patients with a high ECM score group had significantly lower overall survival rates than patients in the low ECM score group. We found that the ECM score was positively associated with immune cells and fibroblasts and negatively correlated with tumor purity. When treated with immunotherapy, BLCA patients with a high ECM score presented a high response rate and better prognosis. We also found that the combination of FSTL1, stage, age, and gender achieved an AUC value of 0.76 in predicting bladder cancer recurrence. Based on the RT-qPCR results of FSTL1 gene expression, there was an overall decrease in the mRNA expression of FSTL1 in cancer tissues compared to their adjacent normal tissues. Subsequent *in vitro* validation demonstrated that the FSTL1 expression was downregulated at the gene and protein level compared to that in SVH cells.

**Conclusion:**

Taken together, our results indicate that ECM-related genes correlate with immune cells, overall survival, and recurrence of BLCA. This study provides a machine learning model for predicting the survival and recurrence of BLCA patients.

## 1. Introduction

Bladder cancer (BLCA) is one of the most common cancers and ranks ninth among all cancers [1]. About 83,730 cases of BLCA were diagnosed in the United States of America in 2021, resulting in 17,200 deaths [2]. The incidence of BLCA increases with patient age [1] and is about four times more common in men than women [3]. Based on the depth of tumor infiltration and stage, BLCA is divided into two categories: non-muscle-invasive (75 percent, NMIBC, Ta, T1, and Cis) and muscle-invasive bladder cancer (25 percent, MIBC, T2-T4) [4]. NMIBC is typically treated with transurethral resection of the bladder tumor (TURBT) plus intravesical chemotherapy [5]. Patients with MIBC are usually treated with radical cystectomy (RC) and pelvic lymphadenectomy [6]. The 5-year survival rate for NMIBC is more than 90% [7]. High recurrence rates (60%) have been observed in NMIBC patients [8], and about 20% of NMIBC patients will progress into MIBC [9]. Concomitantly, approximately half of MIBC patients who receive RC develop recurrences or metastases [7]. The mortality rate at 5 years for MIBC is about 50% [10] with a worsening prognosis for the locally progressive or recurrent MIBC patients. Thus, prediction of the recurrence of BLCA is crucial to the management and therapy.

The development of immune checkpoint inhibitors has been a significant advancement in oncological therapy. Immune checkpoint blockade (ICB) therapies that target PD1 and PDL1 have been approved to treat a variety of malignancies, including melanoma and BLCA [11]. Despite these advancements, only a small percentage of BLCA patients respond to ICB. Reports show that only about 20% of BLCA patients benefit from the treatment with ICB [12]. Given the low response to ICB therapy, some biomarkers such as the expression of PDL1 and immune cells are being considered for detecting potential ICB responders [13, 14]. Presenting another challenge to therapy is the immunosuppressive tumor microenvironment (TME), which impedes the effectiveness of checkpoint inhibitors [15].

The constituents of the TME include blood vessels, immune cells, fibroblasts, and extracellular matrix (ECM). The ECM is a distinct and complex architecture with over 300 proteins within intracellular spaces [16]. The ECM is essential for maintaining tissue homeostasis, and thus, abnormal ECM can promote cancer formation, progression, and metastasis [17]. Accumulation of the ECM in tumors acts as a barrier to block tumor cells from the effects of therapy [18]. Furthermore, the ECM also affects the effectiveness of immunotherapy by preventing immune cells and immunotherapeutic agents from reaching the tumor cell [18]. Cancer patients with highly expressed ECM-related genes have a worse prognosis [19]. Therefore, analysis of the ECM-related genes portends a basis for the prediction of recurrence of BLCA patients.

In this study, we identified six ECM-related genes (CTHRC1, MMP11, COL10A1, FSTL1, SULF1, and COL5A3) that were associated with bladder cancer occurrence, tumor stage, and prognosis based on the bladder cancer tumor samples. These six ECM-related genes were used to construct the ECM score. In addition, we found that ECM scores correlated significantly with tumor stage, tumor prognosis, immune cells, stromal cells, and tumor purity in TME. It has been shown that ECM can be a good prognostic factor for immunotherapy. By RT-qPCR, FSTL1 was significantly reduced in BLCA samples compared to normal bladder samples. Subsequently, *in vitro* validation of the FSTL1 gene and protein expression demonstrated that FSTL1 expression was downregulated in bladder cancer cells compared to that in the normal SV-HUC-1 cell line. Virtual screening and molecular docking of the structures of FSTL1 protein identified three small molecules relevant as predictive molecules. Therefore, the study demonstrates the significance of the ECM as a good prognostic factor for immunotherapy of BLCA patients.

## 2. Materials and Methods

### 2.1. Gene Expression Data Gathering and Processing

Bladder cancer cohorts from the GEO database, including GSE32894 [20], GSE13507 [21], GSE31684 [22], GSE32548 [23], and TCGA-BLCA cohort, were selected and downloaded. For GEO datasets, microarray data profiles were downloaded using the R GEOquery package [24]. Gene expression data (FPKM) of TCGA-BLCA was obtained by the R TCGAbiolinks package [25], which was further transformed into transcripts per kilobase million (TPM). Clinical data, such as survival information, for these BLCA cohorts were collected using the R GEOquery and TCGAbiolinks packages.

### 2.2. Identification of Differentially Expressed Genes (DEGs) and Stage-Associated Genes (SAGs)

To select the genes that are crucial for tumor formation, the DEGs between the 414 tumors and 19 normal bladder tissues from the TCGA-BLCA cohort were identified using the R edgeR package [26]. The genes with “log2FoldChange>1” and “*p* value < 0.05” were considered DEGs. Using the mRNA expression profiles from GSE32894 and TCGA-BLCA, we determined the genes associated with the tumor stage. In order to select the SAGs, a Pearson correlation analysis was conducted (a *p* value of 0.05). The enriched pathways were conducted by the clusterProfiler package [27].

### 2.3. Hub ECM-Related Genes

From the previous study [28], 1026 unique ECM-related genes from 10 ECM gene sets were obtained. A Venn diagram analysis was carried out among the DEGs, SAGs, and ECM-related genes. After that, the intersected genes were further selected by the R package random forest SRC which could calculate the importance value for predicting overall survival (OS) by using the random forest method. The top six genes with the highest importance value were defined as hub ECM genes for bladder cancer occurrence, development, and prognosis.

### 2.4. Calculation of ECM Score

The ECM score was calculated by ECM-related gene expression and coefficients (*β*) from a multivariate Cox regression analysis. ECM score for each bladder cancer sample was determined by the following equation: ECM score = Expression (gene1) × *β* (gene1) + ⋯+Expression (gene6) × *β* (gene6). Bladder cancer samples were split into high and low ECM score groups based on the median ECM score. To estimate the relationships between ECM score/ECM-related hub genes and prognosis, the K-M survival curve analysis based on OS information in the R package survival was used. Besides, the protein values of ECM-related genes were calculated based on the data from the Human Protein Atlas (HPA) data.

### 2.5. Association of ECM Score with TME Cell Populations

The MCP-counter and ssGSEA tools were used to evaluate the proportions of immune cells in each sample [29]. These tools could evaluate the abundance of immune and nonimmune cells by mRNA expression profiles. The ESTIMATE program is capable of generating three types of cell scores: immune score (amount of immune cells), stromal score (amount of stromal cells), and tumor purity (amount of tumor cells) [30].

### 2.6. Association of ECM Score with ICB Response

The IMvigor210 cohort of urothelial cancer patients receiving the anti-PDL1 antibody atezolizumab was selected to predict therapeutic response to ICB [31]. The profiles of mRNA expression data and follow-up information were retrieved from the R package IMvigor210CoreBiologies. By analyzing transcriptome data of bladder cancer patients with the treatment of anti-PD-L1 immunotherapy, we analyzed the association between ECM score and ICB response.

### 2.7. Prediction of Tumor Recurrence in BLCA by Random Forest Classifiers

All the interaction data were randomly separated into the training dataset (70%) and the testing dataset (30%). In the current study, six common machine learning methods were used for model selection, including generalized linear models (GLM), *K*-nearest neighbors (KNN), support vector machine (SVM), and random forest (RF). The area under the curve (AUC), one of the most essential criteria for assessing classification model effectiveness, was selected in the current study. The AUC values of four machine learning algorithms were tested by 5-fold cross-validation in the training dataset, and the machine learning algorithm with the highest AUC value was selected.

### 2.8. Specimen Collection and Preparation

For the FSTL1 gene expression, a total of fifteen bladder cancer specimens and their adjacent normal tissues were obtained from the Second Affiliated Hospital of Henan University of Traditional Chinese Medicine between January and December 2021. All tissues were pathologically diagnosed by two experienced pathologists. Tissue specimens were stored at -80°C for further analysis. Written informed consent was provided by all patients, and all handling was approved by the bioethics committee of our hospital.

### 2.9. Cell Culture

The human immortalized uroepithelial cell line SV-HUC-1 (SVH) and BLCA lines (UMUC-3, T24, SW780, and 5637) were applied in this study. The SVH cell (F-12K medium, Manassas, VA, USA) and other cell lines (RPMI-1640 medium, Gibco, USA) were cultured in the medium supplemented with 10% fetal bovine serum and incubated at 37°C in a 5% CO2 atmosphere.

### 2.10. Gene Expression Analysis

RNA isolation and reverse transcription were described as detailed elsewhere [32]. RT-qPCR was performed with the 7500 Real-Time PCR system (Foster City, CA) relying on the FastStartTM Universal SYBR® Green Master (Roche). Glyceraldehyde 3-phosphate dehydrogenase (GAPDH), *β*2-microglobulin (*β*2M), 18S ribosomal RNA (18S), and *β*-actin were used for normalization by the qbase+ software. The primer sequences are listed in Supplementary Table 1.

### 2.11. Western Blot Analysis

Briefly, the total protein from samples was separated by electrophoresis (10% SDS-PAGE) and transferred onto a polyvinylidene fluoride membrane (PVDF) (Roche, Switzerland). Then, we incubated PVDF membranes with primary antibodies against FSTL1 (1 : 2000, ProteinTech, USA) and *β*-Tubulin (1 : 5000, ProteinTech, USA) at 4°C overnight, followed by washing with TBST and secondary antibodies at room temperature for one hour. Finally, the protein visualization was achieved by an enhanced chemiluminescence (Thermo Fisher Scientific, USA) detection system.

### 2.12. Virtual Screening and Molecular Docking

To discover the potential inhibitors targeting FSTL1, a virtual screening simulation was conducted based on the AutoDock Vina program from the PyRx tool [33]. The protein structure was obtained from the AlphaFold Protein Structure Database [34]. The structures of 1615 FDA-approved small molecules were downloaded from Zinc15 [35]. The AutoDock Vina was set with default configuration parameters in the virtual screening simulation [36]. The top three small molecules having the lowest binding affinity (kcal/mol) to FSTL1 were submitted for molecular docking using AutoDock [37]. Finally, the analysis of binding sites and visualizations were performed using PyMOL programs [38].

## 3. Results

### 3.1. Identification of DEGs and SAGs

**Figure 1:**
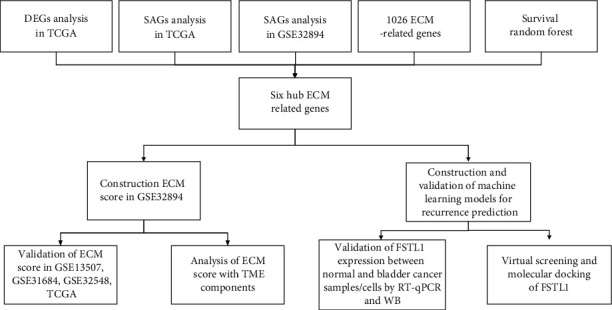
The flowchart of this study.

**Figure 2:**
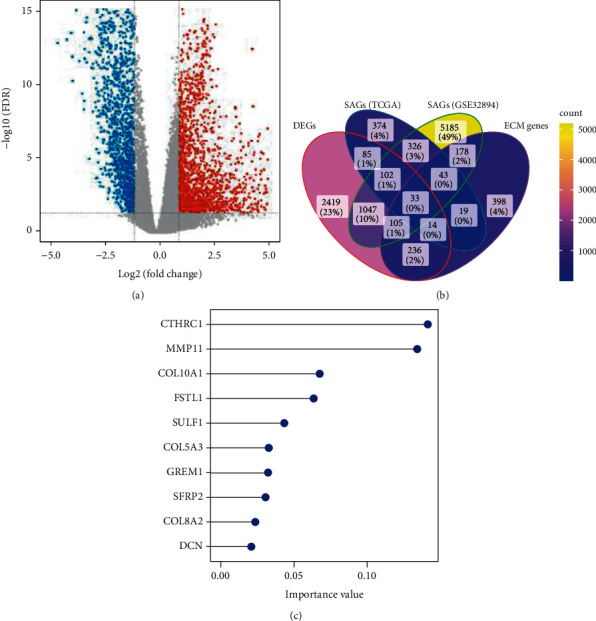
The identification of hub ECM-related genes. (a) Volcano plot for differential gene expression between bladder cancer and normal bladder samples. Red represents upregulation in bladder cancer, blue represents downregulation in bladder cancer, and gray represents no change in expression. (b) Venn diagram of DEGs, SAGs (TCGA), SAGs (GSE32894), and ECM genes. (c) Random forest feature importance ranking for the top 10 ECM genes.

The workflow of our study is shown in Figure 1. Based on the profiles from the TCGA-BLCA cohort which contained 19 normal bladder samples and 414 BLCA samples, we screened out 4041 DEGs (2107 upregulation and 1934 downregulation genes) (Figure 2(a)). In GSE32894, there were 116 (stage 0), 97 (stage 1), and 85 (stage 2) samples. In TCGA-BLCA, there were 130 (stage 2), 140 (stage 3), and 134 (stage 4) samples. Based on pathological stage information, 996 genes from the TCGA dataset and 7019 genes from GSE32894 were found to be significantly associated with the stage (Figure 2(b)). As shown in Supplementary Table 2, “Pathways of ‘Phagosome,” “Graft-versus-host disease,” and “Epstein-Barr virus infection” were found to be enriched in genes associated with the stage from GSE32894. Pathways of “TGF-beta signaling pathway,” “ECM-receptor interaction,” and “choline metabolism in cancer” were found to be enriched in genes associated with the stage from the TCGA-BLCA cohort. Pathways of “dilated cardiomyopathy,” “hypertrophic cardiomyopathy,” and “ECM-receptor interaction” were found to be enriched in DEGs from the TCGA-BLCA cohort. Then, as shown in Figure 2(b), 33 common ECM genes were obtained from the intersection of these 4 groups.

### 3.2. Selection of Hub ECM Genes and Calculation of ECM Score

**Figure 3:**
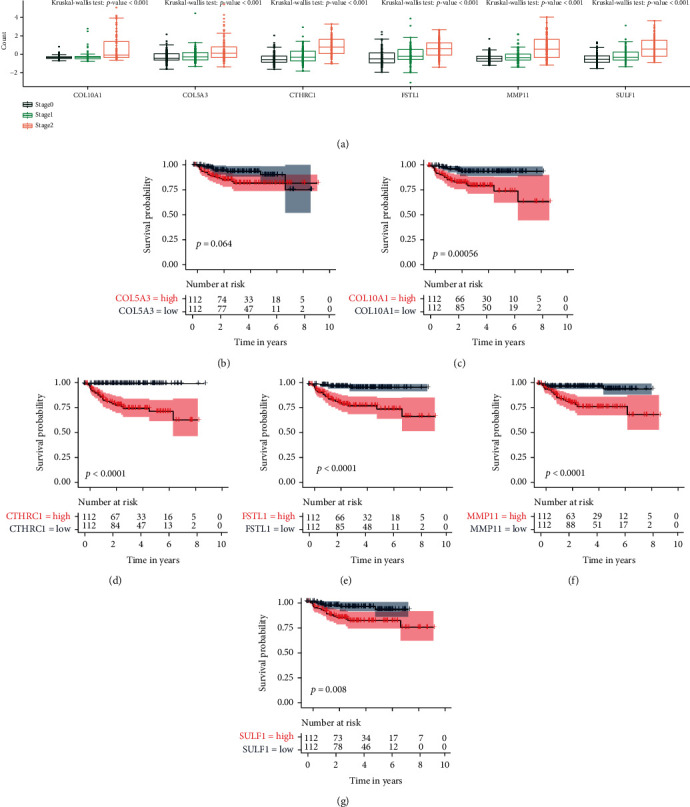
The association of ECM hub genes (COL10A1, COL5A3, CTHRC1, FSTL1, MMP11, and SULF1) with stage and prognosis of bladder cancer of GSE32894. (a) Gene expression of genes in bladder cancer patients according to clinical stage. Analysis of the relationship between (b) COL5A3, (c) COL10A1, (d) CTHRC1, (e) FSTL1, (f) MMP11, (g) SULF1, and bladder cancer survival prognosis based on the Kaplan-Meier.

The intersected genes were further selected by the R package randomForestSRC which can calculate the importance value for predicting overall survival by using the random forest method. As a result, 6 genes were identified: CTHRC1, MMP11, COL10A1, FSTL1, SULF1, and COL5A3 (Figure 2(c)). Based on the TCGA dataset (Supplementary Figure 1), the expression levels of COL10A1, COL5A3, CTHRC1, MMP11, and SULF1 in the bladder cancer tissues were higher than those in the normal bladder tissues. In contrast, the expression quantity of FSTL1 in the bladder cancer tissues was lower than that in the normal bladder tissues. There was a statistically significant difference for these 6 gene expression profiles between different stages (Figure 3(a)). In addition, the higher values of 6 genes were found in the higher stages of BLCA. Survival analysis was used to further evaluate the prognostic significance of these 6 genes. The survival results showed a trend that the lower the expression of COL10A1, CTHRC1, FSTL1, MMP11, and SULF1, the better the OS (*p* value < 0.05) (Figures 3(b)–3(g)). We also did the same analyses on GSE13507 (Supplementary Figure 2), GSE31684 (Supplementary Figure 3), GSE32548 (Supplementary Figure 4), and TCGA-BLCA (Supplementary Figure 5), and the results indicated that these 6 ECM hub genes were deeply associated with tumor stage and prognosis of bladder cancer patients. Among these 6 genes, protein expression data of CTHRC1, MMP11, FSTL1, SULF1, and COL5A3 were available in the HPA dataset (Supplementary Figure 6). CTHRC1, MMP11, and COL5A3 protein levels were increased in bladder cancer than in normal bladder samples (Supplementary Figure 6, Supplementary Table 3). The FSTL1 protein level was lower in bladder cancer than that in the normal bladder samples.

Then, the multivariate Cox proportional hazard model was used to calculate the coefficients of genes in GSE32894. We further established an ECM score model by gene expression values and gene coefficients. The ECM score was calculated for each patient in the training cohort as follows: ECM Score = (3.0026) × Expression (CTHRC 1) + (1.5510) × Expression (MMP 11) + (0.8459) × Expression (COL 10 A 1) + (0.6223) × Expression (FSTL 1) + (−1.2704) × Expression (SULF 1) + ((− 0.7075) × Expression (COL 5 A 3).

**Figure 4:**
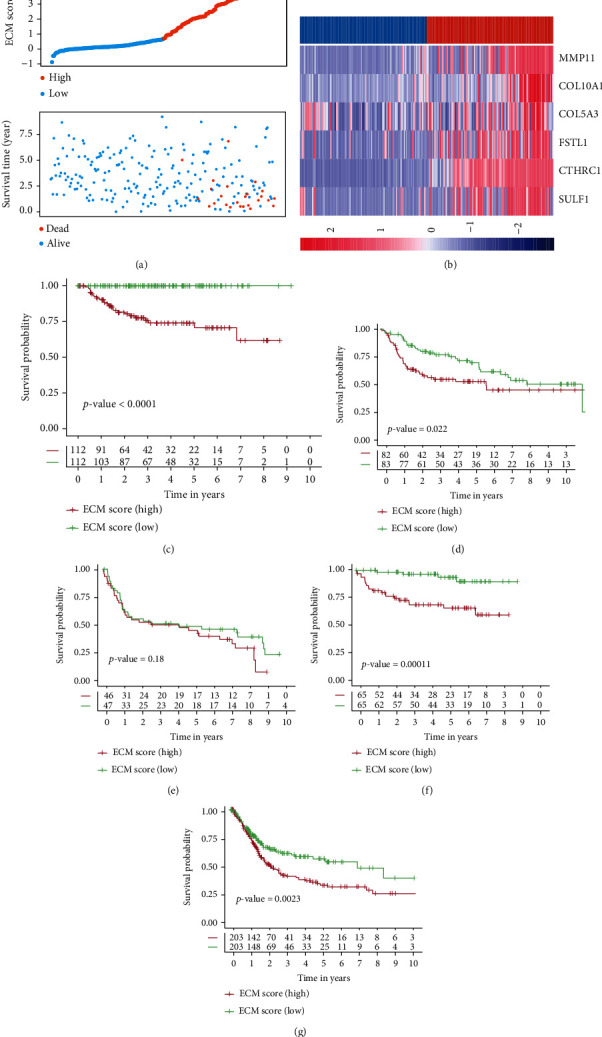
The construction and validation of the ECM score. (a) The distribution and overall survival data were displayed and ranked according to the ECM score. (b) The distribution of gene expression for six ECM genes between low and high ECM score groups. The Kaplan-Meier curves of overall survival time between high and low ECM score groups using the log-rank test in (c) GSE32894, (d) GSE13507, (e) GSE31684, (f) GSE32548, and (g) TCGA-BLCA dataset.

Then we evaluated the ECM score with OS for each patient in GSE32894, GSE13507, GSE31684, GSE32548, and TCGA-BLCA cohorts. The median value of the ECM score was used to distribute samples into the low and high groups. In GSE32894, the ECM index distribution and overall survival data were displayed and ranked according to the ECM score (Figures 4(a) and 4(b)). Patients in the high ECM score group exhibited worse 5-year OS than those in the low ECM score group, as shown in Figure 4(c) (*p* value < 0.0001). In addition, the *p* values of log-rank analysis in GSE13507, GSE31684, GSE32548, and TCGA-BLCA cohorts were calculated and shown in Figures 4(d)–4(g).

### 3.3. TME Cell Populations and Response to Immunotherapies

**Figure 5:**
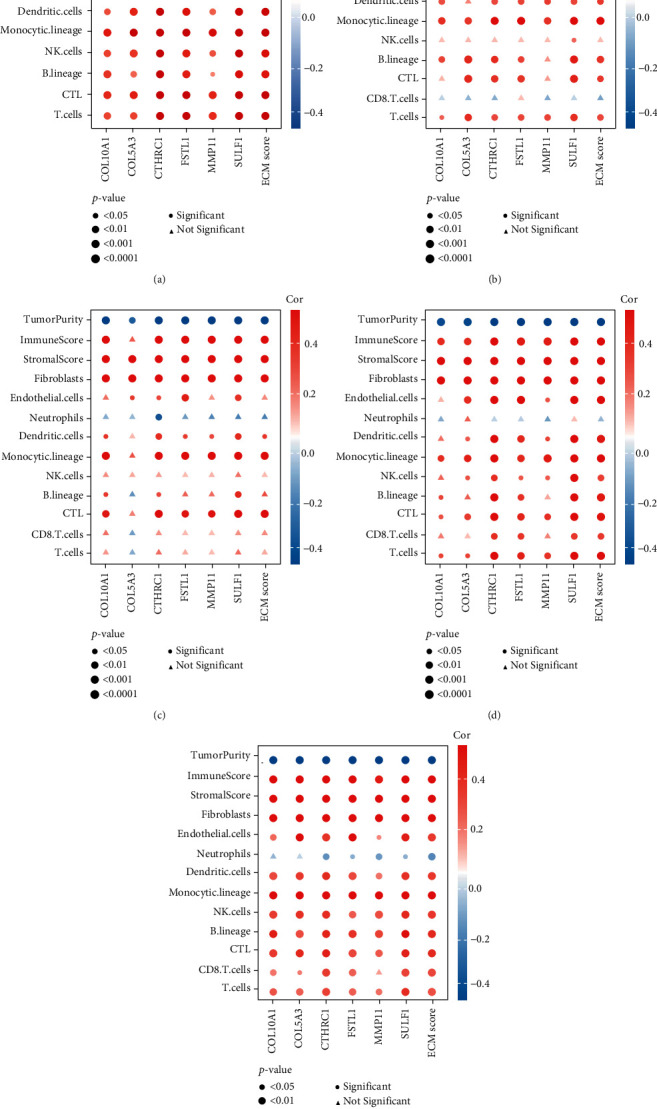
ECM score and hub genes are significantly positively correlated with stromal and immune cell abundance in multiple datasets including (a) GSE32894, (b) GSE13507, (c) GSE31684, (d) GSE32548, and (e) TCGA-BLCA dataset.

In GSE32894, high ECM scores and hub gene expression values were positively associated with several TME cell populations, including stromal and immune cells (Figure 5(a)). In contrast, ECM scores and hub gene expression values were negatively associated with tumor purity (Figure 5(a)). We also performed the same analyses on GSE13507 (Figure 5(b)), GSE31684 (Figure 5(c)), GSE32548 (Figure 5(d)), and TCGA-BLCA (Figure 5(e)), and the results indicated that these 6 ECM hub genes and ECM scores were deeply positively associated with immune and stromal cells and negatively associated with tumor purity of bladder cancer samples.

We also calculated the immune cell values by the ssGSEA method. The results from GSE32894 (Supplementary Figure 7A), GSE13507 (Supplementary Figure 7B), GSE31684 (Supplementary Figure 7C), GSE32548 (Supplementary Figure 7D), and TCGA-BLCA (Supplementary Figure 7E) indicated that these 6 ECM hub genes and ECM scores were deeply positively associated with immune cells.

**Figure 6:**
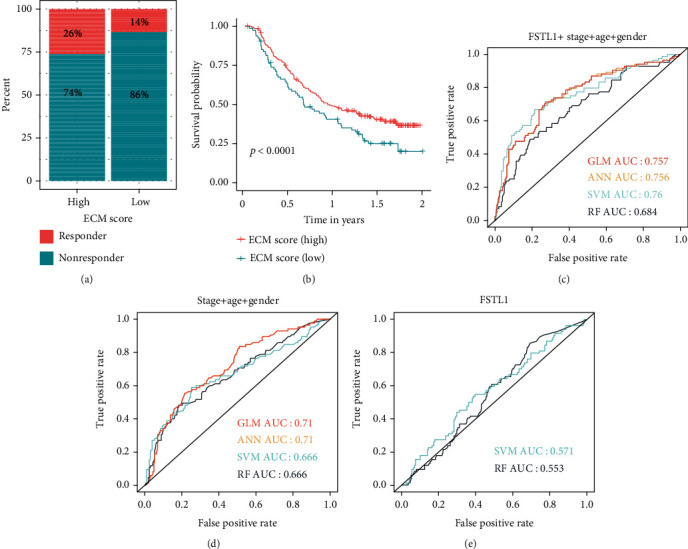
Evaluation of the association of ECM score with drug response to ICB and construction of machine learning models. (a) The correlation of the ECM score with the response rate to immunotherapy in the IMvigor210 dataset. (b) The correlation of the ECM score with the survival analysis in the IMvigor210 dataset. (c) Validation of machine learning models constructed by FSTL1, stage, age, and gender in the testing dataset based on AUC value. (d) Validation of machine learning models constructed by stage, age, and gender in the testing dataset based on AUC value. (e) Validation of machine learning models constructed by FSTL1 in the testing dataset based on AUC value. AUC: area under the receiver operating characteristic curve; RF: random forest.

Then, we determined whether the ECM scores could predict the therapeutic response of bladder cancer patients to ICB treatment. Bladder cancer patients were divided into “responder” and “nonresponder” by the follow-up information, and it was found that the high ECM score group presented a higher percentage of responders (27%) than the low ECM score group (14%) (Chi-squared test: *p* value < 0.01, Figure 6(a)). High and low ECM score groups also had significantly different prognoses for the ICB treatment (Figure 6(b)).

### 3.4. Construction of Machine Learning Models to Predict the Survival of BLCA Patients

Based on recurrence information from GSE32894, GSE13507, GSE31684, and TCGA-BLCA datasets, 675 and 285 samples were defined as nonrecurrent and recurrent patients, respectively. The combination of genes (COL10A1, CTHRC1, FSTL1, MMP11, and SULF1), age, and gender was used to predict the recurrent status of BLCA patients. Firstly, these samples from four datasets (GSE32894, GSE13507, GSE31684, and TCGA-BLCA) were combined into one dataset. Then, the combined dataset was randomly divided into the training (70%) and the testing datasets (30%). In order to reduce the complexity of models, we used the GLM model to rank the importance values of these variables. FSTL1 was selected from these ECM hub genes since it had the highest importance value (Table 1). Then, the commonly used machine learning classifiers including the GLM, ANN, SVM, and RF were constructed to predict the recurrent status of BLCA patients after treatment. After setting the best parameter by a 5-fold cross-validation strategy, we calculated AUC in the testing dataset to characterize the ability of the model to distinguish between nonrecurrent and recurrent cases. The combination of FSTL1 and clinical information demonstrated higher AUC values in predicting recurrent status than using clinical information or FSTL1 alone (Figures 6(c)–6(e)).

### 3.5. Validation of FSTL1 Expression in Bladder Cancer Tissues and Cell Lines

**Figure 7:**
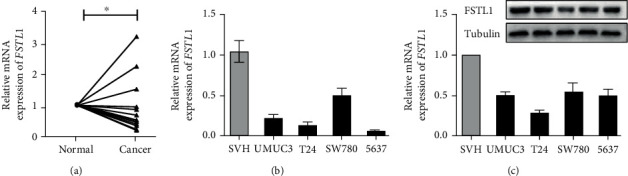
FSTL1 expression was detected in bladder cancer tissues and cell lines. (a) Relative mRNA expression of FSTL1 in fifteen BC tissues and their adjacent normal tissues was examined by RT-qPCR (normalized to *β*-actin). (b, c) The relative expression of FSTL1 in the SV-HUC-1 cell line and four human BC cell lines was tested by (b) RT-qPCR and (c) Western blot. (^∗^*p* value < 0.05, ^∗∗^*p* value < 0.01, ^∗∗∗^*p* value < 0.001, and ^#^*p* value ≥ 0.05). *t*-test was performed to analyze statistical significance. Values are presented as mean ± SEM.

To validate FSTL1 gene expression in BLCA clinical specimens, 15 paired cancer and normal tissue specimens were collected and analyzed using RT-qPCR. As depicted in Figure 7(a), there was an overall decrease in the mRNA expression of FSTL1 in cancer tissues compared to their adjacent normal tissues (*p* value < 0.05). However, 3 cancer tissues presented higher FSTL1 expression. The subsequent *in vitro* validation of the FSTL1 gene and protein expression was carried out in SVH and different BLCA cell lines. The results demonstrated that FSTL1 expression was downregulated in terms of mRNA and protein level compared to that in SVH cells (Figures 7(b) and 7(c)).

### 3.6. Virtual Screening and Molecular Docking

**Figure 8:**
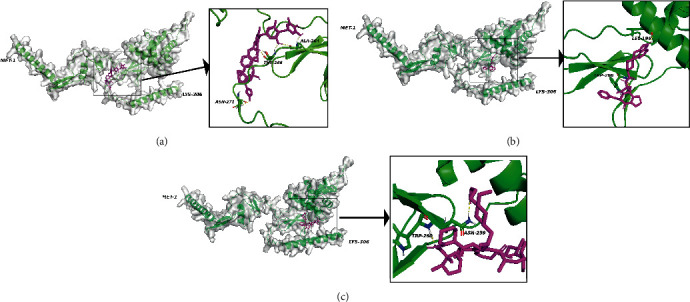
Molecular docking results for ZINC242548690, ZINC3978005, and ZINC169677008. (a) 3D view of the best selected conformation of FSTL1-ZINC242548690. (b) 3D view of the best selected conformation of FSTL1-ZINC3978005. (c) 3D view of the best selected conformation of FSTL1-ZINC169677008. Green color: FSTL1; purple color: small molecules; yellow color: conventional hydrogen bonds.

To find potentially approved drugs that target FSTL1, 1615 FDA-approved small molecules were selected to perform virtual screening. AutoDock VINA from the PyRx tool generated 9 different conformations for each small molecule, which were ranked by binding affinity (kcal/mol). The top 3 ranked small molecules with the lowest binding energy were ZINC242548690 (-9.1 kcal/mol), ZINC3978005 (-8.9 kcal/mol), and ZINC169677008 (-8.9 kcal/mol). The binding sites and interactions between these three small molecules and FSTL1 are displayed in Figures 8(a)–8(c). Based on the 3D view of the best selected conformations, six, four, and two hydrogen bonds were found in the bindings of FSTL1-ZINC242548690 (Figure 8(a)), FSTL1-ZINC3978005 (Figure 8(b)), and FSTL1-ZINC169677008 (Figure 8(c)). The 2D view of the best selected conformations for FSTL1-ZINC242548690 (Supplementary Figure 8A), FSTL1-ZINC3978005 (Supplementary Figure 8B), and FSTL1-ZINC169677008 (Supplementary Figure 8C) were shown.

## 4. Discussion

Bladder cancer (BLCA) is one of the leading causes of cancer morbidity and death worldwide [39]. The high recurrence rate poses a significant challenge in BLCA treatment. Incidentally, most patients with the less invasive NMIBC form of BLCA progress into invasive MIBC that overtly recur and/or metastasize. Metastasis and recurrence of BLCA worsen the prognostic outcome of patients [7]. Thus, it is imperative to predict the recurrence of BLCA based on accurate models. In this current study, we identified six hub ECM genes to construct the ECM score to predict the prognosis of bladder cancer. FSTL1, one of the hub ECM-related genes, was used to construct machine learning models to precisely predict BLCA recurrence and therapy.

The ECM is involved in the general pathogenesis and is therefore a target in cancer therapy. A growing body of evidence reveals that the ECM is an integral part of TME [40, 41]. The ECM is involved in the initiation and development of cancer and is associated with a poor prognosis [42]. A variety of proteins regulating ECM have been linked to the development and poor outcome of BLCA. In the current study, we developed a new ECM score based on six genes (CTHRC1, MMP11, COL10A1, FSTL1, SULF1, and COL5A3). These six genes were observed to correlate positively with tumor stage and negatively correlated with prognosis in bladder cancer samples from GSE32894, GSE13507, GSE31684, GSE32548, and TCGA-BLCA.

Individual studies have similarly reported elevation and/or association of the six selected genes in BLCA. CTHRC1, one of the glycosylated proteins, has been found to be elevated in multiple cancer types and linked to tumor development and metastasis [43]. MIBC presented higher mRNA and protein expression of CTHRC1 than NMIBC, and survival analysis demonstrated that patients with high CTHRC1 were easily exposed to a poor prognosis [43]. MMP11 is a matrix metallopeptidase that regulates ECM proteins. MMP11 expression in BLCA is significantly higher than that in normal bladder tissues [44], and MMP11 expression is positively linked to an aggressive cancer subtype and a poor prognosis in BLCA [45]. COL10A1, one of the collagen family proteins, has been accumulated in tumors, and an earlier study found that COL10A1 was utilized to predict the probability of lymph node metastasis in bladder cancer [46]. FSTL1, a transmembrane extracellular glycoprotein, is a regulator for the expression of ECM molecules [47]. FSTL1 was found to be correlated with a negative prognosis for bladder cancer [4]. SULF1 is one of the regulators of the constituent molecules of the ECM [48], and upregulation of SULF1 is linked to tumor progression and a negative prognosis for bladder cancer [49]. COL5A3 is a member of the collagen family and is associated with metastatic tumor growth [50]. Therefore, the elevation of the six ECM-related genes is linked to the development and metastasis of BLCA.

Infiltration of immune cells is linked to ECM score, according to the findings of ssGSEA. Tumor-infiltrating lymphocytes (TILs), such as activated CD8 T cells, have been linked to a better clinical outcome in a variety of cancer types, including BLCA [51]. In contrast, in the high ECM score group, we found high levels of immunosuppressive cells, such as regulatory T cells (Tregs). Previous studies have shown the association of a high number of Tregs in TME with BLCA recurrence [52]. Therefore, the increased immunosuppressive cells observed in the high ECM score group suggest a reason for the poor prognosis of the patients with high ECM scores. However, the treatment of BLCA patients with PDL1 antibodies in the high ECM group showed a better prognostic outcome than that of the patients in the low ECM score group (Figure 6(b)). Therefore these results suggest that the ECM score could serve as a screening tool for cancer patients to undergo immunotherapy.

Subsequent prediction of BLCA recurrence with FSTL1 and clinical parameters by standard machine learning algorithms, including GLM, ANN, SVM, and RF, demonstrated the utility of machine learning models. Carefully avoiding overfitting situations by using 5-fold cross-validation, we found that the combination of FSTL1, stage, age, and gender achieved the highest AUC value of 0.76 in the testing dataset, which was higher than that of the machine learning models using clinical parameters or FSTL1 alone (AUCs of 0.71 and 0.57, respectively). Thus, the data of FSTL1 and three clinical parameters could accurately predict the recurrence of BLCA using the machine learning model.

In contrast to the five ECM-related genes assessed (CTHRC1, MMP11, COL10A1, SULF1, and COL5A3), BLCA samples/cells had greater levels of FSTL1 mRNA and protein than normal bladder samples/cells (Figure 7, Supplementary Figure 1). The FSTL1 gene is located on chromosome 3q13.33 and consists of 11 exons. The FSTL1 protein structure includes a secretory signal, a follistatin-like domain, a duplicated EF-hand domain, and a VWFC domain [53]. The correlation of FSTL1 with tumorigenesis is complex and disease-specific. For example, FSTL1 has been found to be reduced in a variety of cancer types, including prostate and kidney cancers, but elevated in brain tumors and hepatocellular carcinoma [53]. In our study, we found that FSTL1 was positively associated with fibroblasts and negatively associated with tumor purity (Figure 5). A previous study observed that FSTL1 was expressed in fibroblasts and correlated with metastasis of hepatocellular carcinoma but was barely detectable in cancer cell lines [54]. Therefore, secretion of FSTL1 by primary fibroblasts from normal bladder samples resulted in the expression level of FSTL1 being higher in normal bladder samples than in tumor samples, while the secretion of FSTL1 by cancer-associated fibroblasts (CAF) from the bladder tumors increases the carcinoma malignancy and decreases the prognosis of bladder cancer.

The antitumor potential of digoxin, dihydroergotamine, and everolimus is being researched in cancer therapy [55–60]. Digoxin (ZINC242548690), a cardiac glycoside, has recently been proposed as one of the new therapeutic drugs for cancers [55]. The results of the cell function assays revealed that digoxin inhibited the proliferation and migration of lung cancer cells [55]. It also could significantly inhibit the growth of pancreatic cancer cells [56] and inhibit primary tumor growth and the metastasis of breast cancer [57]. Dihydroergotamine (ZINC3978005) is an ergot alkaloid medicine that is used to treat a migraine or cluster headache attack. Findings demonstrate that dihydroergotamine can induce lung cancer cell death by promoting apoptosis and mitophagy [58]. Everolimus (ZINC169677008) is one of the mTOR inhibitors and has been approved for the treatment of multiple cancers such as breast cancer and renal cell carcinoma [59]. A phase II study demonstrated that it possesses meaningful antitumor activity in a subset of patients with advanced bladder cancer [60].

Despite the significant findings of the study, we identify potential drawbacks. First, in the dataset, only 19 normal bladder tissues were identified in the TCGA database and were not enough for a broader comparative assessment. Second, the relationship between the ECM score and the immune cell infiltration has not been thoroughly studied. Third, only FSTL1 expression values of mRNA and protein were validated by RT-qPCR and Western blot. Therefore, future experiments will increase the sample size of the dataset and investigate the relationship between the ECM score, ECM-related genes, and immune cells and the correlation between FSTL1 and BLCA clinical parameters.

## 5. Conclusion

The challenge in the management and prognosis of BLCA could be addressed by ECM-related genes using a machine learning model. We identified six ECM-related genes that are better correlated with tumor stage and prognosis. A high ECM score is also associated with immune cells in BLCA, demonstrating a better response rate to immunotherapy. The ECM score could therefore be a promising predictive biomarker for the response to immunotherapy in BLCA. Machine learning models could predict the recurrence of BLCA patients. Therefore, this study contributes to a better understanding of the relationship between ECM and TME cells and offers a way to predict bladder cancer recurrence.

## Figures and Tables

**Table 1:** The importance values in the GLM machine learning model.

Variables	Importance value
FSTL1	100
Grade	83.79657422
MMP11	66.20959301
Stage	47.83572557
SULF1	42.50328061
COL10A1	16.70044387
Age	0.59890661
Gender	0
COL5A3	0
CTHRC1	0

## Data Availability

The datasets used in this study are available from the corresponding author on reasonable request.

## Supplementary Materials

Supplementary Materials Table S1: sequences of primer. Table S2: Encyclopedia of Genes and Genomes (KEGG) analysis of differentially expressed genes (DEGs) and stage-associated genes (SAGs) from GSE32894 and TCGA-BLCA. Table S3: the information of immunohistochemistry (IHC) images from the Human Protein Atlas (HPA) dataset. Figure S1: comparison of gene expression values of COL5A3 (A), COL10A1 (B), CTHRC1 (C), FSTL1 (D), MMP11 (E), and SULF1 (F) in bladder cancer and normal bladder samples. Figure S2: the association of ECM hub genes (COL10A1, COL5A3, CTHRC1, FSTL1, MMP11, and SULF1) with the stage and prognosis of bladder cancer of GSE13507. (A) Gene expression of genes in bladder cancer patients by clinical stage. Analysis of the relationship between COL5A3 (B), COL10A1 (C), CTHRC1 (D), FSTL1 (E), MMP11 (F), and SULF1 (G) and bladder cancer survival prognosis based on the Kaplan-Meier. Figure S3: the association of ECM hub genes (COL10A1, COL5A3, CTHRC1, FSTL1, MMP11, and SULF1) with stage and prognosis of bladder cancer from GSE31684. (A) Gene expression of genes in bladder cancer patients according to clinical stage. Analysis of the relationship between COL5A3 (B), COL10A1 (C), CTHRC1 (D), FSTL1 (E), MMP11 (F), SULF1 (G), and bladder cancer survival prognosis based on the Kaplan-Meier. Figure S4: the association of ECM hub genes (COL10A1, COL5A3, CTHRC1, FSTL1, MMP11, and SULF1) with stage and prognosis of bladder cancer from GSE32548. (A) Gene expression of genes in bladder cancer patients according to clinical stage. Analysis of the relationship between COL5A3 (B), COL10A1 (C), CTHRC1 (D), FSTL1 (E), MMP11 (F), SULF1 (G), and bladder cancer survival prognosis based on the Kaplan-Meier. Figure S5: the association of ECM hub genes (COL10A1, COL5A3, CTHRC1, FSTL1, MMP11, and SULF1) with stage and prognosis of bladder cancer from GSE32548. (A) Gene expression of genes in bladder cancer patients according to clinical stage. Analysis of the relationship between COL5A3 (B), COL10A1 (C), CTHRC1 (D), FSTL1 (E), MMP11 (F), SULF1 (G), and bladder cancer survival prognosis based on the Kaplan-Meier. Figure S6: Protein expression values of COL5A3, CTHRC1, FSTL1, MMP11, and SULF1. Immunohistochemistry images of COL5A3 in the normal bladder (A) and the bladder cancer samples (B). Immunohistochemistry images of CTHRC1 in the normal bladder (C) and the bladder cancer samples (D). Immunohistochemistry images of FSTL1 in the normal bladder (E) and the bladder cancer samples (F). Immunohistochemistry images of MMP11 in the normal bladder (G) and the bladder cancer samples (H). Immunohistochemistry images of SULF1 in the normal bladder (I) and the bladder cancer samples (J). Figure S7: ECM score and hub genes are significantly positively correlated with stromal and immune cell abundance in multiple datasets including GSE32894 (A), GSE13507 (B), GSE31684 (C), GSE32548 (D), and TCGA-BLCA (E) dataset. Figure S8: the 2D view of the best selected conformations for FSTL1-ZINC242548690 (A), FSTL1-ZINC3978005 (B), and FSTL1-ZINC169677008 (C) were shown.
