# Supplementary material for: Prediction of Prognosis and Recurrence of Bladder Cancer by ECM-Related Genes
Source: J Immunol Res. 2022 Apr 12;2022:1793005. doi: 10.1155/2022/1793005 (PMC9018183; doi:10.1155/2022/1793005)
Supplement: Supplementary Materials — Table S1: sequences of primer. Table S2: Encyclopedia of Genes and Genomes (KEGG) analysis of differentially expressed genes (DEGs) and stage-associated genes (SAGs) from GSE32894 and TCGA-BLCA. Table S3: the information of immunohistochemistry (IHC) images from the Human Protein Atlas (HPA) dataset. Figure S1: comparison of gene expression values of COL5A3 (A), COL10A1 (B), CTHRC1 (C), FSTL1 (D), MMP11 (E), and SULF1 (F) in bladder cancer and normal bladder samples. Figure S2: the association of ECM hub genes (COL10A1, COL5A3, CTHRC1, FSTL1, MMP11, and SULF1) with the stage and prognosis of bladder cancer of GSE13507. (A) Gene expression of genes in bladder cancer patients by clinical stage. Analysis of the relationship between COL5A3 (B), COL10A1 (C), CTHRC1 (D), FSTL1 (E), MMP11 (F), and SULF1 (G) and bladder cancer survival prognosis based on the Kaplan-Meier. Figure S3: the association of ECM hub genes (COL10A1, COL5A3, CTHRC1, FSTL1, MMP11, and SULF1) with stage and prognosis of bladder cancer from GSE31684. (A) Gene expression of genes in bladder cancer patients according to clinical stage. Analysis of the relationship between COL5A3 (B), COL10A1 (C), CTHRC1 (D), FSTL1 (E), MMP11 (F), SULF1 (G), and bladder cancer survival prognosis based on the Kaplan-Meier. Figure S4: the association of ECM hub genes (COL10A1, COL5A3, CTHRC1, FSTL1, MMP11, and SULF1) with stage and prognosis of bladder cancer from GSE32548. (A) Gene expression of genes in bladder cancer patients according to clinical stage. Analysis of the relationship between COL5A3 (B), COL10A1 (C), CTHRC1 (D), FSTL1 (E), MMP11 (F), SULF1 (G), and bladder cancer survival prognosis based on the Kaplan-Meier. Figure S5: the association of ECM hub genes (COL10A1, COL5A3, CTHRC1, FSTL1, MMP11, and SULF1) with stage and prognosis of bladder cancer from GSE32548. (A) Gene expression of genes in bladder cancer patients according to clinical stage. Analysis of the relationship between COL5A3 (B), COL10A1 (C), CTH [file 1793005.f1.zip › Supplementary Table 1.docx]

| Gene name | Primer sequence |
| --- | --- |
| FSTL1 | Forward：5´-TCTGTGCCAATGTGTTTTGTGG-3´ |
|  | Reverse：5´-TGAGGTAGGTCTTGCCATTACTG-3´ |
| GAPDH | Forward：5'-ACAGTCAGCCGCATCTTCTT-3' |
|  | Reverse：5'-GACAAGCTTCCCGTTCTCAG-3' |
| B2M | Forward：5'-GATGAGTATGCCTGCCGTGT-3' |
|  | Reverse：5´-TGCGGCATCTTCAAACCTCC-3´ |
| β-actin | Forward：5´-CTCGCCTTTGCCGATCC-3´ |
|  | Reverse：5´-GAATCCTTCTGACCCATGCC-3´ |
| 18S rRNA | Forward：5´-CGGCGGAAAATAGCCTTTGC-3´ |
|  | Reverse：5´-TCTGGATCTTGTACTGGCG-3´ |

**Supplementary Table 1.** Sequences of Primer.
